# Roles of three putative salmon louse (*Lepeophtheirus salmonis*) prostaglandin E_2_ synthases in physiology and host–parasite interactions

**DOI:** 10.1186/s13071-021-04690-w

**Published:** 2021-04-19

**Authors:** Sussie Dalvin, Christiane Eichner, Michael Dondrup, Aina-Cathrine Øvergård

**Affiliations:** 1 Institute of Marine Research, SLCR-Sea Lice Research Centre, Nordnes, P. box 1870, 5817 Bergen, Norway; 2 Department of Biological Sciences, SLCR-Sea Lice Research Centre, University of Bergen, P. box 7803, 5020 Bergen, Norway; 3 Department of Informatics, SLRC-Sea Lice Research Centre, University of Bergen, P. box 7803, 5020 Bergen, Norway

**Keywords:** Invertebrate, Arthropod, Copepod, Blood-feeding, RNA interference, ROS, Immune response

## Abstract

**Background:**

The salmon louse (*Lepeophtheirus salmonis*) is a parasite of salmonid fish. Atlantic salmon (*Salmo salar*) exhibit only a limited and ineffective immune response when infested with this parasite. Prostaglandins (PGs) have many biological functions in both invertebrates and vertebrates, one of which is the regulation of immune responses. This has led to the suggestion that prostaglandin E_2_ (PGE_2_) is important in the salmon louse host–parasite interaction, although studies of a salmon louse prostaglandin E_2_ synthase (*PGES*) *2* gene have not enabled conformation of this hypothesis. The aim of the present study was, therefore, to characterize two additional PGES-like genes.

**Methods:**

*Lepeophtheirus salmonis* microsomal glutathione *S*-transferase 1 like (*LsMGST1L*) and *LsPGES3L* were investigated by sequencing, phylogenetics, transcript localization and expression studies. Moreover, the function of these putative PGES genes in addition to the previously identified *LsPGES2* gene was analyzed in double stranded (ds) RNA-mediated knockdown (KD) salmon louse.

**Results:**

Analysis of the three putative *LsPGES* genes showed a rather constitutive transcript level throughout development from nauplius to the adult stages, and in a range of tissues, with the highest levels in the ovaries or gut. DsRNA-mediated KD of these transcripts did not produce any characteristic changes in phenotype, and KD animals displayed a normal reproductive output. The ability of the parasite to infect or modulate the immune response of the host fish was also not affected by KD.

**Conclusions:**

Salmon louse prostaglandins may play endogenous roles in the management of reproduction and oxidative stress and may be a product of salmon louse blood digestions.

**Graphic Abstract:**

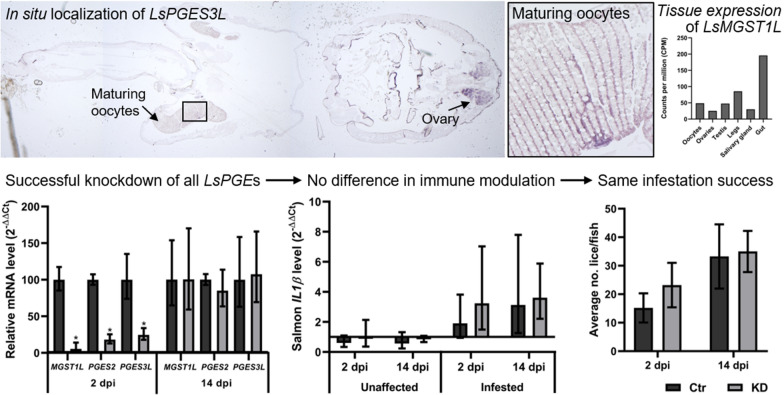

**Supplementary Information:**

The online version contains supplementary material available at 10.1186/s13071-021-04690-w.

## Background

The salmon louse (*Lepeophtheirus salmonis*) is a marine ectoparasite of salmonid fish, feeding on fish mucus, skin and blood [1, 2]. Salmon lice have a life-cycle consisting of eight stages, starting with three planktonic larval stages: nauplius 1, nauplius 2 and the infective copepodid stage. All further development takes place on the fish and consists of two chalimi, two preadult stages and the adult stage [3, 4]. Heavy infestations can cause severe problems to the host, including wounding, secondary infections and osmotic disturbances [5, 6]. Infestations of farmed fish have been difficult to manage due to the development of resistance against parasiticides [7], and the negative impact on wild salmonid fish through transmission of lice from farmed to wild fish stocks is a cause of environmental concern [8–10].

Prostaglandin E_2_ (PGE_2_) is an eicosanoid component with hormone-like functions, derived from arachidonic acid (AA) through three sequential enzymatic reactions [11]. Generally, phospholipase A_2_ releases AA from cell membranes, a cyclooxygenase (COX) converts AA to PGG_2_ and further to PGH_2_, which can be metabolized to PGE_2_ by PGE_2_ synthases (PGESs). The action of PGE_2_ is dependent on the binding of prostanoid EP receptor subtypes (EP1–4), and PGE_2_ has diverse roles in physiological processes, such as inflammation, reproduction and embryonic development, depending on cell type and EP receptor subtype [12–16].

PGESs are found in a large range of organisms where they play diverse physiological roles [17]. In mammals, three isotypes of PGES exist, two of which are membrane bound (PGES1 and -2) and one cytosolic (PGES3). These isotypes typically display different enzymatic properties, expression patterns, cellular localizations and functions. PGESs have also been described in many arthropods, including crustacean species, and similar to mammals, three genes encoding PGESs have been found in penaeid shrimp [18]. The functional property of a few invertebrate synthases has also been confirmed, where the conversion of PGH_2_ to PGE_2_ by recombinant *Gammarus* and *Caprella* sp. PGES2 has been demonstrated [19]. PGESs and their product PGE_2_ are involved in reproduction, including maturation of ovaries and vitellogenesis in prawn and crabs [18, 20–22]. PGES has also been detected in blood cells of crab, indicative of a role in immunity [23], and PGE_2_ has been detected in both tapeworms and crab nervous tissue where it is likely to be important for development and function [20, 24].

Parasites with a long-term interaction with their host need mechanisms to evade potential host immune reaction, and prostaglandins (PGs) such as PGE_2_ have been found to play important roles in many host–parasite protozoan and metazoan interactions [25, 26]. However, uncovering the role of PGs is complicated by their diverse roles and the fact that these compounds are produced by both the host and the parasite [27]. In blood-feeding arthropods such as ticks, PGs are suggested to be involved in vasodilation and immune modulation in the host, as high concentrations of PGE_2_ are found in tick saliva [28–30]. Both PGE_2_ and PGD_2_ have been detected in gland secretions from the fish tapeworm *Diphyllobothrium dendriticum* when incubated in serum from a host fish [24], and* in vitro* studies have suggested that PGE_2_ derived from the tapeworm *Schistocephalus solidus* have the potential to modulate host leukocyte viability and reactive oxygen species production [31].

Atlantic salmon (*Salmo salar*) display a limited and mainly local immune response to salmon louse, which does not eliminate the infection [32–35]. This has led to efforts to understand how the salmon louse modulates its host, and PGE_2_ has been put forward as a putative candidate for immune modulation. PGE_2_ has been detected in excretory/secretory products from dopamine-treated salmon louse, and it was demonstrated that the level of PGE_2_ quickly dropped after lice were removed from the host fish [36, 37]. Moreover, Atlantic salmon immune tissue express the EP4 prostaglandin E receptors [38], and PGE_2_ treatment has been shown to down-modulate the expression of MHC class I proteins and interleukin-1β in a salmon macrophage-like cell line [37]. However, in a more recent study in which excretory/secretory products were obtained by agitating lice in sea water, PGE_2_ was not identified [39]. Moreover, studies of a putative salmon louse gene, *PGES2* (*LsPGES2*) were unable to reveal any involvement of LsPGES2 in the host–parasite interaction [40]. Instead, expression of *LsPGES2* indicated a function in reproductive organs, although no visible alteration in reproductive phenotype was observed in knockdown (KD) animals.

In the present study, we hypothesized that this lack of phenotypic alterations is due to the action of additional salmon lice PGES genes beyond the already described* LsPGES2* and that these may be involved in the host–parasite interaction in addition to endogenous processes. Two additional genes with putative PGES activity were identified and their functional role analyzed by double-stranded (ds) RNA-mediated KD and studies of host–parasite interactions, reproduction, oxidative stress and blood digestion.

## Methods

### Culture of salmon lice and source of tissue

Laboratory strains of the Atlantic salmon lice subspecies *Lepeophtheirus salmonis salmonis* [41] were maintained on farmed Atlantic salmon (*Salmo salar*) according to Hamre et al. [42]. The salmon were hand fed a commercial diet and reared in sea water with a salinity of 34.5 g/kg and a temperature of 10 °C. Eggs, nauplii and copepodids were kept in seawater from the same source. Nauplii were obtained from hatching eggs and kept in a flow-through single-well system [42].

For ontogenetic expression analysis of the two PGES-like genes, *LsMGST1L* and *LsPGES3L*, we collected a time-series of all lice stages from egg to adult lice, in pentaplicate samples, as follows: (i) eggs: 1 egg sac (string) (containing approximately 200 eggs; (ii) nauplius 1, nauplius 2 and copepodids (free-living) (approximately 100 larvae); copepodids, 2 days post-infestation (dpi) and 4 dpi (60 larvae); (iv) chalimus: chalimus 1 (30 animals) and chalimus 2 (20 animals); (v) preadult and adult stages: single animals (adult females were defined as young before the first egg extrusion had taken place and as mature when producing eggs).

To analyze transcripts during the reproductive cycle, adult females were collected in RNAlater® (Qiagen, Hilden, Germany) while their egg strings were removed and kept in a flow-through single-well system at 10 °C [42]. Adult female lice continuously produce eggs that are deposited in batches approximately every 10 days at 10 °C. After deposition of the eggs carried externally by the female until hatching, a new batch of eggs (300–700) is matured and undergoes vitellogenesis in the genital segment [43]. To determine where in the reproductive cycle the sampled females were at sampling, the hatching day of the egg strings after sampling was recorded. The sampled females were further grouped according to days prior to hatching of their egg strings.

To analyze PGE_2_ levels in adult females in relation to blood-feeding, adult female lice were removed from a host fish and either frozen directly on dry ice or kept in saltwater in flow-through single-well incubators [42] for 3 h after removal from the host at 10 °C before sampling on dry ice. The animals were kept at − 80 °C until further analysis.

### RNA purification and cDNA synthesis

All samples for RNA isolation were collected in RNAlater® (LifeTechnologies, Carlsbad, CA, USA), kept at 4 °C overnight and stored at − 20 °C. Total RNA was isolated with a combined TRI Reagent® (Sigma-Aldrich, St. Louis, MO, USA) and RNeasy (Qiagen) method, as previously described [44]. Extracted total RNA was either frozen at − 80 °C or used directly for cDNA synthesis. For real-time reverse transcriptase (RT)-PCR, cDNA synthesis was carried out using the AffinityScript qPCR cDNA Synthesis Kit (Stratagene, San Diego, CA, USA) with oligo(dT) as primers according to the supplier recommendations, adding 200 ng lice total RNA or 1000 ng salmon total RNA. The cDNA samples were diluted five times and stored at − 20 °C until use.

To produce a template for PCR, the qScript cDNA SuperMix (Quanta BioSciences, Gaithersburg, MD, USA) was used, applying 1 µg total RNA from adult female salmon louse.

### PCR, cloning and sequencing

Candidate PGES genes were identified within the salmon louse genome by BLAST search in LiceBase (www.LiceBase.org). Gene-specific primers were designed (Table 1), and rapid amplification of cDNA ends (RACE; (5′ and 3′) was performed using the SMARTer™ RACE cDNA amplification kit (Clontech Laboratories, Inc. Mountain View, CA, USA) using 1 μg total RNA from adult female lice.

**Table 1:** Primers used for rapid amplification of cDNA ends, in situ hybridization, double-stranded RNA synthesis for knockdown studies and quantitative PCR of the target gene and reference gene for *Lepeophtheirus salmonis* and *Salmo salar*

Gene^a^	Forward primer (5′-3′)	Reverse primer (5′-3′)	Use^b^
LsMGST1	GGAATTAATGACGATTTATGGGCTAGT	TGTCATTGAGATGCGCTCTTCTTACAC	R, IS, K
LsMGST1	TTTCGCCAATCAAGAGGACGCCAA	GGGCGCAATGAGAAGAAAGGGGACT	qPCR
LsPGES2	AAAGGTGGATCATTTGGTGGCGG	CGAGAGCTCACAGCCCATTTCATCTT	qPCR
LsPGES3L	CCAAGGTGATTTGGGCTCAAACCG	TCCTTTCCGCCCACGCCACTAA	qPCR
LsPGES3L	ATGGAGCTCTTCGACGAGGTCGTT	CCCCGCTATCAGATTGGTCCAAG	R, IS, K
LsEF1α	GGTCGACAGACGTACTGGTAAATCC	TGCGGCCTTGGTGGTGGTTC	qPCR ref
LsADT3	CTGGAGAGGGAATTTGGCTAACGTG	GACCCTGGACACCGTCAGACTTCA	qPCR ref
Ls18s	GCAGCAGGCACGCAAATT	GATGAGTCCGGCTTCGTTATTTT	qPCR ref
SsC3a	ATTCTTCCCCTCCACTCCCTCG	CGATTTGGTCGTCAAGCCAGG	qPCR
SsIL1β	GCTGGAGAGTGCTGTGGAAGA	TGCTTCCCTCCTGCTCGTAG	qPCR
SsIL4/13a	CGTACCGGCAGCATAAAAATCACCATTCC	CCTTGCATTTTGTGGTGGTCCCA	qPCR
SsIL8	GCATCAGAATGTCAGCCAGCC	ACGCCTCTCAGACTCATCCC	qPCR
SsIL10	ATGAGGCTAATGACGAGCTGGAGA	GGTGTAGAATGCCTTCGTCCAACA	qPCR
SsTNFα	CACTGCCACCAAGAGCCAAG	CGCCAGTTGTCATCGCATACC	qPCR
SsIFNγ	ATGGATGTGTTATCAAGGGCTGTGATGTG	CAGCTGGTCCTTGGAGATCTTATAGTGGAC	qPCR
SsMHC2	GGACGTGAGGTGAAGTCTGATGTGACC	CTGATGTGCTCCACCATGCAGGA	qPCR
SsCD4	GAGTACACCTGCGCTGTGGAAT	GGTTGACCTCCTGACCTACAAAGG	qPCR
SsCD8α	TAGAGTGCAAGACAACGCTGGAATGGA	TCTCGAGCCTTTTTGAAAGCCTTCAG	qPCR
SsCD8β	CAGCCTCAACAATGCTGACCGGACCTA	GCGTCCTCTGCCGTTATATTATTGATGC	qPCR
SsNCCRP1	AATCCTGCGCCTCACGGTGTGAGTC	GCGAGGAGGTCCTTCTGGTGGAAAC	qPCR
SsIgM	TGAGGAGAACTGTGGGCTACACT	TGTTAATGACCACTGAATGTGCAT	qPCR
SsIgT	GGTGGTCATGGACGTACTATTT	CCTGTGCAGGCTCATATCTT	qPCR
SsEF1α	CACCACCGGCCATCTGATCTACAA	TCAGCAGCCTCCTTCTCGAACTTC	qPCR ref
SsTRIM	TTACTGTAGGAGCTGTATTGAGGGCTGCTG	TTCTCCACCAGCTCAGCCAACATG	qPCR ref

^a^Ls, *Lepeophtheirus salmonis*; Ss, *Salmo salar*

^b^R, RACE (rapid amplification of cDNA ends ); IS, in situ hybridization, K , knockdown; qPCR, quantitative PCR of target gene; qPCR ref., quantitative PCR of reference gene

The RACE products were further cloned using the TOPO TA Cloning® Kit for sequencing (Invitrogen, Carlsbad, CA, USA). Colonies were used as templates for the PCR reaction with M13 forward and reverse primers, and the PCR products were further purified by ExoSAP-it (Affymetrix; Thermo Fisher Scientific, Waltham, MA, USA) prior to sequencing. The purified PCR products were sequenced using the BigDye Terminator v3.1 Cycle Sequencing Kit from Applied Biosystems and run by an ABI PRISM 7700 automated sequencing detecting system (Applied Biosystems, Foster City, CA, USA) at the University of Bergen sequencing facility.

### Bioinformatic analysis

Sequences were assembled and translated using Vector NTI Advance 10 software (Invitrogen). The open reading frame (ORF) was blasted using ExPASy BLAST form (http://web.expasy.org/blast/) and NCBI blast form (http://blast.ncbi.nlm.nih.gov/Blast.cgi), and aligned in Clustal Omega (http://www.ebi.ac.uk/Tools/msa/clustalo/). Location of domains was predicted by InterPro (http://www.ebi.ac.uk/interpro/). Searches for orthologous sequences to PGE synthases were conducted using BlastP in the predicted *L. salmonis* proteome in Ensembl Metazoa (release 48) and TBlastN against the representative LSalAtl2s genome assembly (https://metazoa.ensembl.org/Lepeophtheirus_salmonis/Info/Index). Blast searches were executed both on the command line under Linux (NCBI Blast suite version 2.9.0+), Ensembl Metazoa, and through licebase.org using LsPGES2 (EMLSAP00000000441) and human and *Drosophila melanogaster* mGST and PGES-like protein sequences as queries (UniProtKB accessions: Q9H7Z7, O14684, Q15185, B3KPZ2, Q8SY19, Q9V420) with a Blast E-value < 1e−6. In addition, we searched Ensembl (release 101) and Ensembl Metazoa for precomputed orthologues of *D. melanogaster* and *L. salmonis* having GO:0050220 (PGES activity) as Gene Ontology (GO) term annotation or InterPro annotation IPR023352 (Membrane associated eicosanoid/glutathione metabolism-like domain superfamily).

The expression of the three putative *PGES* was also compared to high-throughput data from previous investigations. For tissue-specific analysis, the expression measured by microarray of five different tissues (intestine female and male, ovary, testis, subcuticular tissue and frontal neuronal and gland enriched tissue) [45] was used. For each of the three genes, one oligo was shown (EMLSAT00000000441: CUST_3823_PI425513912; EMLSAT00000006733: CUST_21227_PI425553006; EMLSAT00000012943: CUST_11144_PI425553006), as identified in LiceBase GBrowse. For investigation of expression changes during development with respect to molting, RNA sequencing data from a series of chalimus and preadult 1 larvae of different instar age with respect to molting were used [46]. In this study, chalimus 1, 2 and preadult 1 salmon louse larvae were divided by size measurements into different sex and age groups (young, middle, old and molt, according to their instar age). The young group included lice that had recently molted to the respective stage; the middle group included lice approximately in the middle of the stage; the old group contained lice that were about to molt to the next stage; and the molt group contained the lice showing morphological signs of molting.

### Phylogenetic analysis

Orthologous amino acid sequences for EMLSAP00000006733 and EMLSAP00000012943 were obtained by Blast searches* versus* UniprotKB and GeneBank [47, 48]. Sequence alignments and accessions used in the analysis are provided in Additional files 1 and 2. Multiple sequence alignments (MSA) were computed using MUSCLE via the MUSCLE web-service with default parameter [49]. MSAs were inspected and manually end-clipped to the first and last conserved column using Jalview [50]; no further editing of MSAs was applied. Phylogenetic trees were constructed using the Markov Chain Monte Carlo method (MCMC) in MrBayes (v3.2.6) with 5 million generations, eight chains and four rate categories for the Gamma distribution [51]. RAxML (v8.2.9) was used for maximum likelihood (ML) analysis with 1000 bootstrap iterations [52]. Substitution models for ML analysis were determined by prottest3 for each set of orthologous sequences based on the two best fit model under the Akaike information criterion (AIC) [53]; these were LG + I + Γ + F and LG + Γ + F for both groups of orthologues. MrBayes was first applied in mixed mode, and the resulting best fit model was selected for a second MCMC run, always allowing for invariant sites, resulting in the WAG + I + Γ model for both groups. Phylograms were inspected and rendered in FigTree v1.4.4 (http://tree.bio.ed.ac.uk/software/figtree/) and figures were edited in Inkscape.

### *In situ* hybridization

Salmon lice were fixed in 4% paraformaldehyde in 4% phosphate buffer (pH 7.4) for 24 h at 4 °C, and the specimens were then processed in the Reichert-Jung Histokinette 2000 rotary tissue processor (Leica Microsystems GmbH, Wetzlar, Germany) where they were washed in phosphate-buffered saline, dehydrated through a graded ethanol series and embedded in paraffin wax. Sections, 4.0 μm thick, were cut with a Leica RM 225 microtome (Leica Microsystems). Digoxigenin (DIG)-labeled antisense and sense RNA probes of the two putative *PGES* were prepared by* in vitro* transcription using the DIG RNA Labeling Kit (Roche AG, Basel, Switzerland) on purified PCR products (Table 1) that included T7 promoters in the templates.* In situ* hybridization was performed according to [54], with some modifications as earlier described [55]. Hybridizations with sense probes were carried out as negative control.

### Real-time PCR

Real-time PCR was performed and the products analyzed as described below using the established salmon louse elongation factor 1 alfa (*eEF1α*) and adenine nucleotide translocator 3 (*ADT3*) gene as reference genes when analyzing developmental stages and animals on which RNA interference (RNAi) had been performed [56, 57]. In animals where RNAi was performed targeting *LsHPX1* (see next section),* 18S* was used as reference gene as *eEF1α* and *ADT3* previously have been found to be affected by *LsHPX1* KD [57]. Primers used for real-time RT-PCR are listed in Table 1. Real-time PCR was performed with the 1× PowerUp™ SYBR Green Master Mix (Thermo Fisher Scientific), 500 nM forward and reverse primers and 2 µl diluted cDNA in 10-µl reaction volumes. Samples were run in duplicate on the Applied Biosystems 7500 Real-Time PCR System under standard conditions (50 °C, 2 min; then 95 °C/2 min; 95 °C/15 s and 60 °C/1 min for 40 cycles; followed by a melt curve analysis at 60–95 °C. A five-point standard curve of fourfold dilutions was made for each assay to calculate PCR efficiencies, given by the equation *E*% = (10^1/slope^ − 1) × 100 [58]. The relative differences in quantification cycle (Cq) between the target gene and the reference genes (ΔCq) and expression relative to a calibrator (ΔΔCq) were calculated, transformed by the equation 2^−ΔΔCq^ [59]. T-tests were used to determine differently expressed genes with a threshold *P* value of 0.05.

### RNA interference

Genes selected for KD were analyzed to identify sequences suitable for specific KD by treatment with dsRNA. Primers were chosen within regions that showed little similarity to other salmon louse genes. Moreover, the chosen fragment was aligned against the salmon louse genome by BlastN to exclude similarity between DICER-produced 19-mers and any salmon louse gene. RNAi was performed as previously described in nauplius [60]. In short, long dsRNA for the CPY control gene (cod trypsin) and PGES genes were produced using the Ambion MEGAscript® RNAi Kit (Thermo Fisher Scientific) according to supplier’s instructions using the primers listed in Table 1. Additionally, dsRNA targeting *LsHPX1* transcripts was performed to create animals with increased levels of oxidative stress [57]. For soaking of nauplius, pools of 20–60 nauplius 1 larvae from the same egg string were incubated in 10 ng/µl dsRNA for each gene and incubated overnight (17 h). Thereafter, all animals from the same treatment group were pooled and transferred to a flow-through well, where they were kept until sampling.

For RNAi in preadult 2 females, a modified protocol based on [61] was used. Before injection of dsRNA (600 ng/ul), 1 μl of saturated and filtered bromophenol blue was added to 50 μl dsRNA solution; this concentration of bromophenol blue has been found to be well tolerated by the salmon louse [61]. The needle was placed dorsally into the haemocoel of the cephalothorax close to the ovaries. The custom-made needles were pulled by utilizing a 1-mm Borosilicate glass tube with an inner diameter of 0.5 mm (Sutter Instrument, Novato, CA, USA) on the P-2000 laser-based micropipette puller system (Sutter Instrument). The dsRNA solution was added to the needle using a microloader tip and then coupled to a HI-7 injection holder (Narishige Ltd., London, UK). The tip of the needle was removed with a scalpel blade. By blowing air into the needle, less than 1 µl solution was injected and quickly spread in the louse, as observed by the dispersal of blue color. After injection and a brief 2-h period in seawater, the lice were placed back onto fish to observe their development to adult individuals and measure their reproductive output. The gene expression was analyzed in control and KD animals.

Photographs were taken of lice from the RNAi experiments using a Canon EOS 600D camera (Canon Inc., Tokyo, Japan) mounted with an adaptor (LMscope) to an Olympus SZX9 dissecting microscope (Olympus Corp., Tokyo, Japan).

### Infection trials

Copepodites treated with dsRNA targeting control fragments or a combination of the putative *PGES* genes were used to infect fish that were kept in single tanks (six fish/treatment group). Lice sampled to analyze KD by real-time RT-PCR were harvested at 2 and 14 dpi of fish, when the lice were still copepodites or had developed into the preadult 1 stage, respectively. From the fish, two different sample types of salmon skin, including the dermis, epidermis and scales, were collected on the same days: one sample was taken directly under a louse and one from nearby non-parasitized skin. Skin was obtained only from areas of the fish with scales since distinct expression of Atlantic salmon immune genes after salmon lice infestation has been observed in scaled skin compared to scaleless skin on the head region [62]. Moreover, skin at or above the lateral line was avoided to ensure more homogenous samples among individuals. The immune response in skin samples was analyzed with the primers listed in Table 1.

### Prostaglandin E_2_ and F_2α_ analysis

The Prostaglandin E Metabolite EIA Kit and the 13,14-dihydro-15-keto Prostaglandin F2α ELISA Kit (Cayman Chemical Company, Ann Arbor, MI, USA) were used to measure the levels of PGE_2_ and PGF_2α_ metabolites in LsHPX1 KD copepodids and adult lice homogenates, according to supplier’s instructions with some modification as follows. For the PGE_2_ analysis, around 200 copepodids were pooled or one female was homogenized in 75 µl ddH_2_O using 1.4-mm zirconium oxide beads (Precellys 24; Thermo Fisher Scientific) and a TissueLyser LT (Qiagen) for 4 min at 50 Hz. Samples were centrifuged for 5 min at 1.5 *g*, and 55 µl of the supernatant was added to 16.5 µl carbonate buffer. After an overnight incubation on 37 °C, 22 µl of phosphate buffer and 16.5 µl EIA buffer were added, and 50 µl of the sample was added to two wells (two technical replicates). For the PGF_2α_ analysis, around 150 copepodids were pooled in 100 µl ddH_2_O (*N* = 3) and homogenized as described above. Samples were centrifuged for 5 min at 1.5 *g*, and 50 µl of the supernatant was added to the ELISA wells. Two technical replicates were run for each of the three biological replicates.

## Results

### Sequence analysis

Searches in the salmon louse genome (www.licebase.org) revealed two putative PGE synthases in addition to the already published *LsPGES2* [40]. Based on similarity to other genes, the putative *PGES* were named *Lepeophtheirus salmonis* microsomal glutathione *S*-transferase 1 like (*LsMGST1L*) and prostaglandin E synthase 3 like (*LsPGES3L*).

The sequenced *LsMGST1L* cDNA sequence (Accession no. MW495051) consists of 607 bp, containing an ORF of 432 bp followed by a 135-bp 3′-untranslated region (UTR). The ORF translates into a 146-amino acid (aa) protein identified as a microsomal glutathione* S*-transferase 1 like (MGST1) protein holding three transmembrane helixes. No other proteins within the membrane-associated proteins in eicosanoid and glutathione metabolism (MAPEG) superfamily were found within the salmon louse genome. Blast searches (BlastP, NCBI) with the LsMGST1L aa sequence showed the highest resemblance to genes annotated as MGST1 and PGES1 in both arthropod and vertebrate species. The PGE_2_ synthase activity of human PGES1 is glutathione dependent [63], and amino acids important for glutathione binding and enzymatic activity [64, 65] were found to be conserved in LsMGST1L.

The *LsPGES3L* cDNA sequence (Accession no. MW495052) consists of 952 bp, containing an ORF of 537 bp and a 305-bp 3′-UTR. The ORF translates into a 179-aa protein containing an alpha crystalline-Hsps-p23 like superfamily domain, predicted to have a cytosolic localization. Blast searches (BlastP, NCBI) with LsPGES3L showed the highest resemblance to genes annotated as prostaglandin E synthase 3 from arthropods, especially ants and termites, but also vertebrates and even mammalian PGES3.

### Phylogenetic analysis

**Fig. 1:**
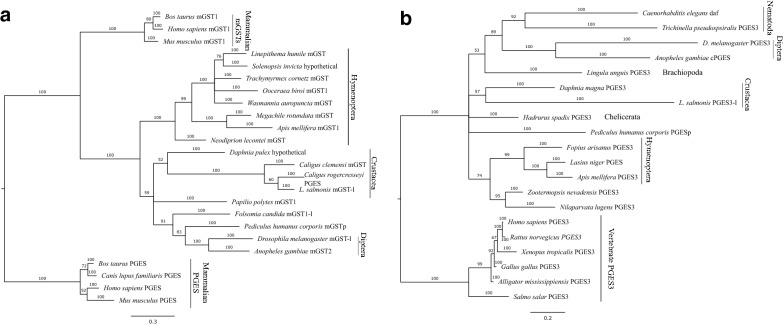
Phylogenetic trees for orthologues to EMLSAP00000006733 (MGST1L) (**a**) and EMLSAP00000012943 (PGES3L) (**b**). Branch labels indicate branch support in percentage, scale bars correspond to 0.3 (**a**) and 0.2 (**b**) substitutions per site. Sequence annotations are given as found. Both phylograms were rooted using mammalian (**a**) and vertebrate (**b**) sequences as outgroups. Both salmon lice sequences are well embedded within taxa with the same annotation, except for *Caligus rogercresseyi* PGE_2_ synthase (*PGES*). Major clades (Hymenoptera, Diptera, Nematoda and Crustacea) are indicated by vertical bars

Using Bayesian (Fig. 1) and ML phylogenies (Additional file 3: Figure S1), we assessed whether homologous protein sequences of genes similar to PGES were clustered correctly with homologous arthropod sequences of similar annotation. Both phylogenies reconstructed large arthropod clades and vertebrate outgroups correctly, even though for some branches MrBayes was unable to determine bifurcation. ML analysis by RAxML produced bifurcations, but with low branch support. Overall, the ML phylogenies were less consistent with species relationships. Both, mGST-like and PGES3-like sequences largely form clades with sequences of the same annotation, and salmon louse sequences are well embedded within clades of closely related taxa. The only outlier with respect to annotation is *Caligus rogercresseyi* PGES, which is the sister taxon to LsMGST1L and is embedded in a cluster of mGST-like sequences. Notably, all Bayesian phylograms reproduce the major taxonomic groups that contain multiple sequences, such as Crustacea, into which the salmon louse is embedded. As expected, deeper branches indicate higher sequence diversity in invertebrates than in vertebrates.

### Developmental expression of *LsPGESs*

**Fig. 2:**
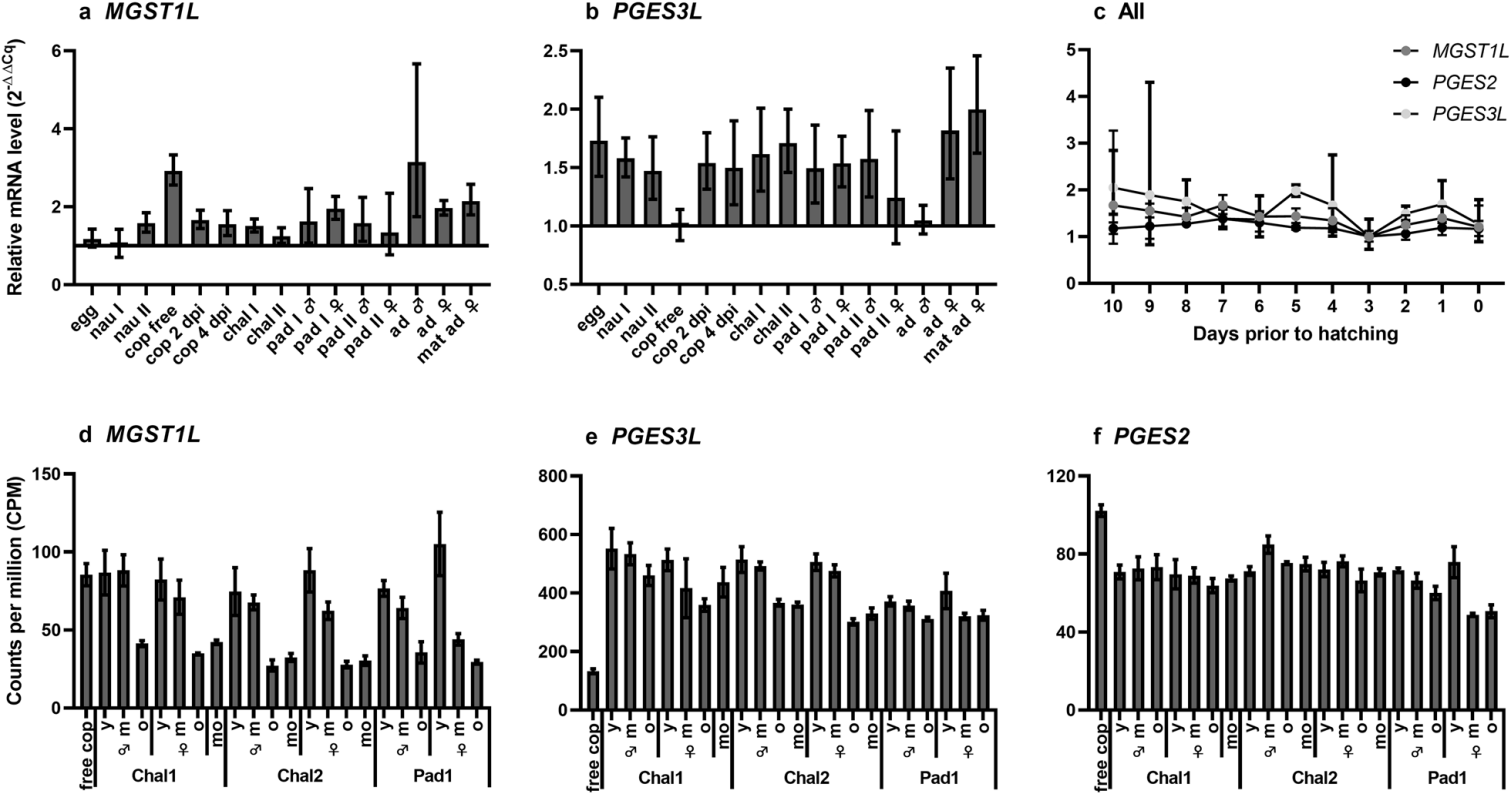
Transcriptional profile of *LsMGST1L*,* LsPGES2* and *LsPGES3L* during development measured by qPCR (**a**–**c**) and RNA sequencing (**d**–**f**). **a**, **b** Transcription level of *LsMGST1L* and *LsPGES3L* in all life stages: egg (fertilized), nauplius (*nau*), copepodites (*cop*), planktonic copepodites (*cop free*), cop days post-infestation of fish (*cop pdi*), chalimus (*cha*), readult (*pad*), adult (*ad*) and mature adult (*mat ad*). Expression is given as average 2^−∆∆Cq^ ± standard deviation (SD) (N = 5). **c** Transcription of *LsMGST1L*,* LsPGES2* and *LsPGES3L* during the reproductive cycle of adult females given as average 2^−∆∆Cq^ ± SD (*N* = 5). The *x*-axis indicates the number of days before eggs carried by the female will hatch and new egg extrusion will take place. **d**–**f** Detailed ontogenetic expression profile during the chalimus (*Cha1*,* Chal2*) and preadult 1 (*Pad1*) stages: *LsMGST1L* (**d**),* LsPGES3L* (**e**) and *LsPGES2* (**f**). Data are presented as average counts per million (CPM) ± SD (*N* = 3).* y* Young,* m* middle,* o* old,* mo* molting

*LsMGST1L* and *LsPGES3L* transcript levels were determined in all salmon louse developmental stages (Fig. 2a, b). Constitutive expression of both genes was detected throughout lice development, with small variations observed between stages. *LsMGST1L* did not show a transcriptional response to the shift from free-living stages to parasitic stages or major differences between male and females. Expression of *LsPGES3L* was similar, except for low levels of transcription in planktonic copepodites and adult males. These results were confirmed by inspection of transcriptional patterns based on RNA sequencing (Additional file: S1; Source: Licebase.org, EMLSAG00000006733 and EMLSAG00000012943).

The expression patterns of all three *LsPGES gene*s were also investigated during the reproductive cycle of adult females (Fig. 2c) and in a more detailed ontogenetic analysis of animals with different stage-age (Fig. 2d–f). Analysis of *LsPGES* transcripts during the three molt cycles of chalimus 1, 2 and preadult 1 revealed different patterns of expression. Whereas *LsPGES2* was expressed at similar levels throughout the molt cycle of chalimi and the preadult 1 stage, *LsMGST1L* exhibited a cyclic expression pattern. Higher levels of *LsMGST1L* were detected in newly molted lice and lice in the middle of the stage, while lower levels were detected in lice directly before the next molt. This cyclic pattern was also evident for *LSPGES3L*, especially in the expression in chalimus 2 larvae.

Adult female lice produce eggs continuously that are deposited in batches extruded into egg strings shortly after hatching of the previous batch. At 10 °C, this reproductive cycle lasts approximately 10 days [43]. To further determine the involvement of the putative LsPGES genes in reproduction, the transcriptional levels were analyzed during an entire reproductive cycle. The *LsPGES* transcript levels were, however, not found to change significantly during this cycle (Fig. 2c), although a large variation in the expression of *LsPGES3L* was evident in females directly after egg string extrusion.

### Tissue specific expression of *LsPGESs*

**Fig. 3:**
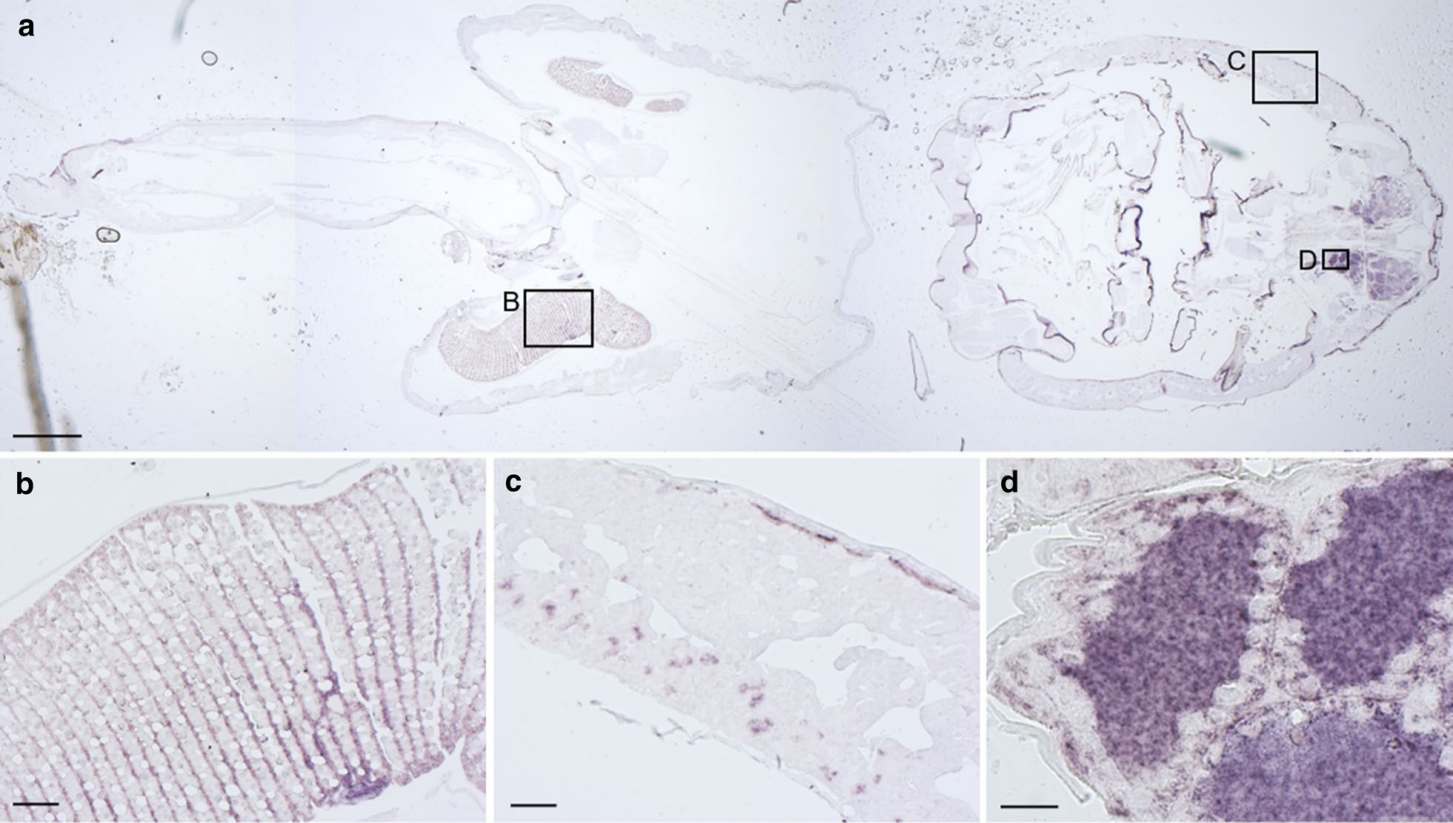
Localization of *LsPGES3* by in situ analysis of an adult female salmon louse (*Lepeophtheirus salmonis*). Dark coloring indicates the presence of transcripts. **a** Overview of the louse with positive staining seen in ovaries, oocytes and subepidermal tissue. Boxes denoted **B**, **C** and **D** indicate the position of the high magnification photos. Scale bar (**a**): 500 μm. **b** Unfertilized eggs in genital segment; scale bar: 50 μm. **c** Subepidermal tissue; scale bar: 50 μm. **d** Ovary; scale bar: 20 μm

To investigate the localization of *LsMGST1L* and *LsPGES3L* in salmon louse tissues, we performed* in situ* hybridization in sections of adult females. *LsPGES3* transcripts were detected in the ovaries and developing oocytes within the genital segment and in some cells of the subepidermal tissue (Fig. 3). Parallel sections were incubated with the sense probe as a negative control, which displayed only unspecific staining of the cuticula (results not shown).

**Fig. 4:**
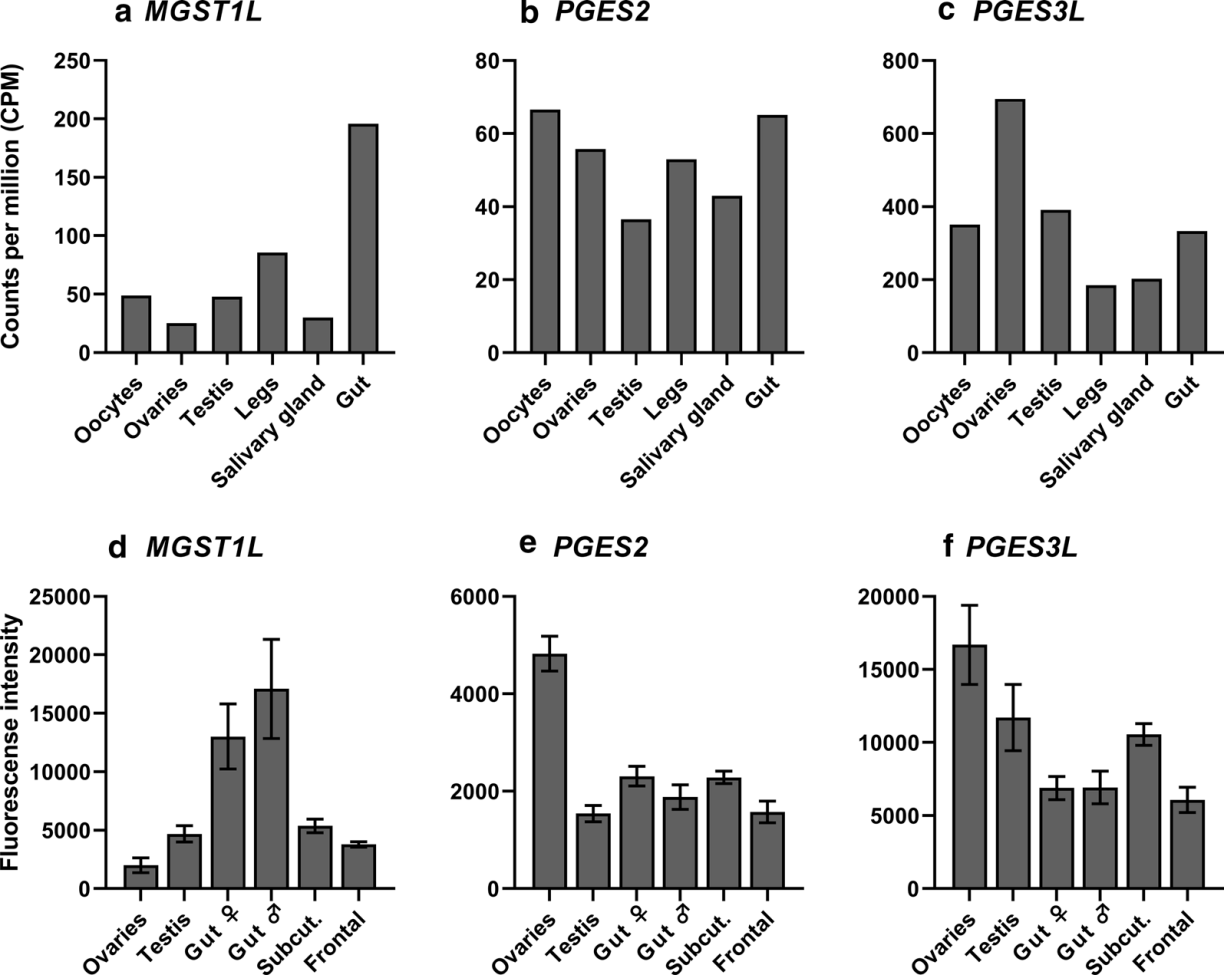
mRNA level of the three putative *PGES* genes in different tissues/body segments analyzed by RNA sequencing (*N* = 1) (**a**–**c**) and microarray (*N* = 4) (**d**–**f**). Error bars indicate standard deviation

Probably due to lower levels of *LsMGST1L* in individual cells, *LsMGST1L* was not successfully localized by* in situ* hybridization. Therefore, we analyzed the transcript level in the RNA sequencing data from a range of tissues and body segments (Source: Licebase.org, EMLSAG00000012943, EMLSAG00000000441 and EMLSAG00000006733) and in previously published microarray data [45]. *LsPGES2* and *LsPGES3L* exhibited a similar pattern of expression, with the highest transcript level detected in the ovaries, especially in the RNA sequencing data (Fig. 4). *LsMGST1L* exhibited a slightly different pattern of expression, with the highest transcript level detected in louse intestine. Moreover, none of the genes showed high expression in the sample with a high content of salivary gland or in the leg sample where tegmental type 1–3 glands are present [66].

### PGES KD in larvae

To investigate the functional role of LsPGESs in salmon lice, RNAi was induced in nauplius 1 larvae. KD was performed using dsRNA fragments targeting *LsMGST1l*, *LsPGES2* and *LsPGES3L*, and the effects on the animals were evaluated as the nauplii developed to the infective copepodid stage. Visual inspection of the animals revealed no abnormal morphology and pattern of movement or molting abnormalities compared to control animals (results not shown).

**Fig. 5:**
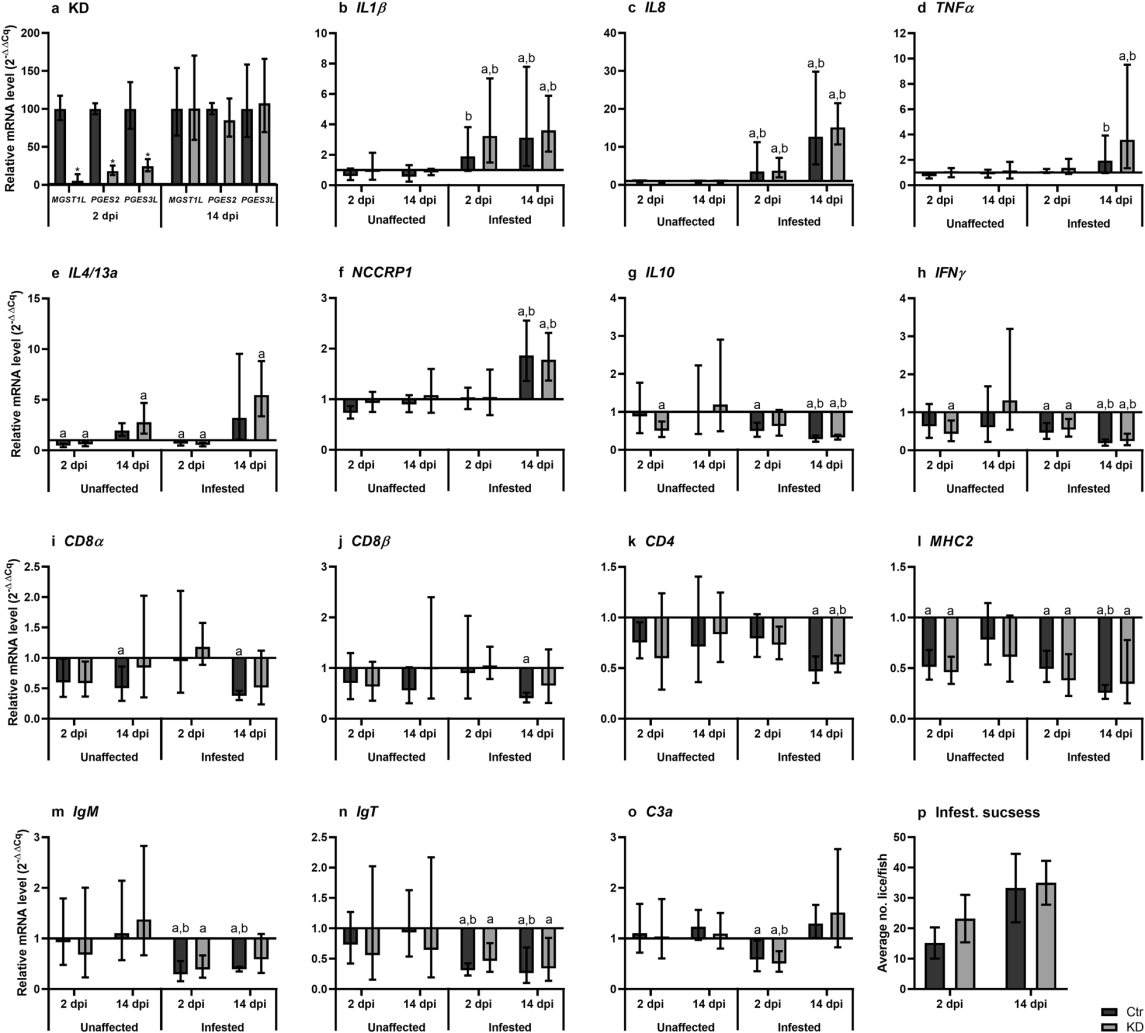
**a** Knockdown (KD) of *MGST1L*,* PGES2* and *PGES3L* in copepodids at 2 and chalimus 2 at 14 dpi. Expression level in KD lice were related to that of control lice (2^−ΔΔCq^ ± SD, *N* = 4). Asterisk denotes significant KD compared to control lice. **b**–**o** mRNA level of selected immune genes in salmon (*S. salar*) skin infested with control and KD lice (*L. salmonis)*. Skin was sampled 2 and 14 dpi at the site of lice attachment (infested) and in unaffected sites. The expression level was calculated as 2^−∆∆Cq^ ± SD (*N* = 5) related to untreated control fish. Lowercase letters above bars indicate statistical significance from the untreated control fish (*a*) and from unaffected skin (**b**). **p** Salmon louse infestation success after KD, given as average number of lice/fish ± SD. *IL* Interleukin,* TNF* tumor necrosis factor,* NCCRP* non-specific cytotoxic cell receptor P, *IFN* interferon,* CD* cluster of differentiation,* MHC* major histocompatibility complex,* Ig *immunoglobulin,* C* complement

Due to the suggested role of lice-derived PGE_2_ in the immunomodulation of susceptible salmonid hosts, *LsMGST1L*, *LsPGES2* and *LsPGES3L* KD copepodids were used to infest fish with subsequent analysis of the local immune response. At 2 dpi, the levels of all three transcripts were significantly reduced by 95% (*LsMGST1L*), 82% (*LsPGES2*) and 75% (*LsPGES3L*) compared to control animals, but at 14 dpi when the lice were in the chalimus 2 stage, they had returned to the level of the control animals (Fig. 5a). Sampling of fish skin was performed in parallel with sampling of the lice, and the expression of immune-related transcripts was analyzed. The transcript levels of the cytokines *interleukin* (*IL*)*1β*, *IL4/13a* and *IL8* and of the *non-specific cytotoxic cell receptor P1* (*NCCRP-1*) in skin samples was significantly increased, especially at 14 dpi at the site of infestation compared to skin sections taken away from the louse (unaffected) and untreated control fish (Fig. 5b–o). Transcripts of T-cell markers *cluster of differentiation 4* (*CD*4), *CD8α*, *CD8β*, *major histocompatibility complex 2* (*MHC2*) and *immunoglobulin T *(*IgT*) were all expressed at a lower level when compared to untreated fish. Similar to the trend observed in upregulated transcripts, the response was stronger and—for some genes—only significant at 14 dpi. There were, however, no significant differences between control and KD lice for any of the analyzed genes, and infestation success was also similar between the two groups (Fig. 5p).

### PGES KD in preadult and adult stages

To investigate the functional role of the putative *PGES* genes in mobile lice, RNAi was induced in preadult 2 females and followed during their development to adults carrying their second egg string. KD was performed using two mixtures of dsRNA fragments targeting only *LsPGES2* and *LsPGES3L* (RNAi1) or all three putative *PGES*s (RNAi2). Even though *LsMGST1l*,* LsPGES2* and *LsPGES3* were significantly reduced in KD animals (t-test, *P* < 0.001), all injected lice were successful at re-establishing on the host fish and underwent further development at a normal rate (Table 2). Visual inspection and morphological measurements of lice revealed no significant differences in survival on host fish, egg production, size or morphology compared to control lice (t-test, *P* > 0.05). Due to the mobile nature of preadult and adult lice, local immune responses were not assessed.

**Table 2:** Overview of interference RNA experiment injecting preadult 2 female salmon louse (*Lepeophtheirus salmonis*)

RNAi experiments^a^	Expression (% of control)			Recovery^b^	Length^c^ (mm) ± SD	
	*LsMGST1l*	*LsPGES2*	*LsPGES3L*		Total	Egg string
Control	100	100	100	14/30	1.10 (± 0.05)	2.19 (± 0.30)
RNAi1	n/a	60	58	20/30	1.13 (± 0.06)	2.27 (± 0.27)
RNAi2	37	63	48	20/30	1.12 (± 0.03)	2.24 (± 0.20)

RNAi, RNA interference; SD standard deviation

Gene expression and morphological measurements were performed as lice developed to the adult stage

^a^Knockdown was performed in females treated with dsRNA fragments targeting *LsPGES2* and *LsPGES3L* (RNAi1 group), and in females treated with dsRNA fragments targeting *LsMGST1l*, *LsPGES2* and *LsPGES3L.* (RNAi2 group)

^b^Recovery indicates the number of adult females found on fish compared to the number of injected preadult 2 females placed on fish

^c^Total length is the average length of the recorded females, including cephalothorax, genital segment and abdomen. Egg string length is the average length of egg strings carried by gravid females (all females carried eggs)

### Prostaglandin E_2_ and blood feeding

**Fig. 6:**
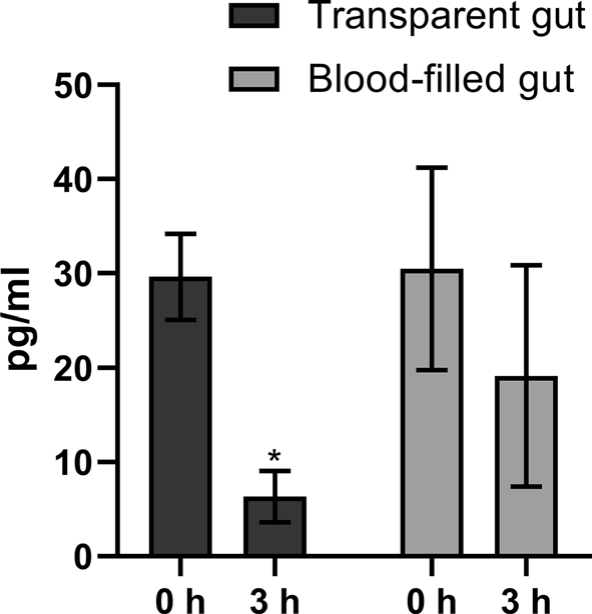
Indirect measurement of the prostaglandin E_2_ (PGE_2_) level (mean ± SD, *N* = 5) in adult lice with transparent or blood-filled gut directly after sampling (0 h) and at 3 h post-sampling. Asterisk indicates a significant difference in the amount of PGE_2_ metabolites, determined by t-tests with a threshold *P* value of 0.05

Prostaglandin E_2_ in parasitic lice can either originate from an endogenous source or from the fish blood or skin. To determine whether the content of PGE_2_ in the louse was affected by blood-meal digestion, the amount of PGE_2_ metabolites was measured in adult females. Measurements were performed in four groups: females with blood-filled gut and females with a transparent gut with no visible blood; these were subdivided into one group measured just after collection from the fish (0 h) and one group measured 3 h later (Fig. 6). At 0 h, the amount of PGE_2_ metabolites was equal in both groups (with or without blood). After 3 h, significantly lower levels (fivefold decrease) of PGE_2_ metabolites were detected in females with transparent guts. A decrease in the amount of PGE_2_ metabolites from 0 to 3 h was also observed in females with a blood-filled gut, but this decrease was smaller and not significant.

### Oxidative stress and expression of *LsMGST1L*, *LsPGES2* and *LsPGES3L*

**Fig. 7:**
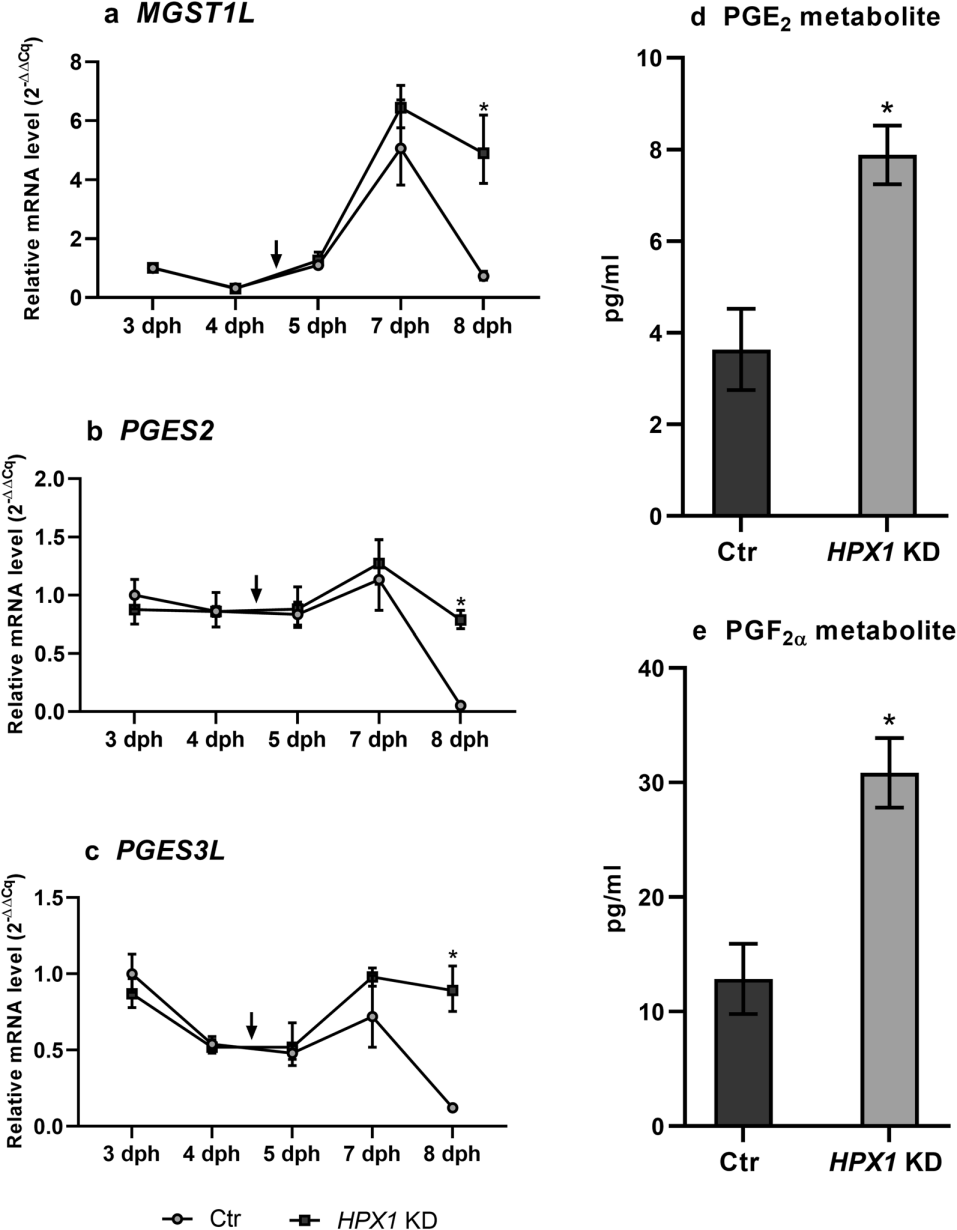
**a**–**c** Relative mRNA level of the three putative *PGES* genes in control and *LsHPX1* KD animals. Expression was calculated relative to* 18S* expression, and the average mRNA level ± SD is shown (*N* = 3). Arrows indicate the approximate time of molting from nauplius 2 to copepodids. **d** Analysis of PGE_2_ metabolites.** e** PGF_2α_ metabolites in control and KD animals at 8 days post-hatching (*dph*). Average metabolite amount ± SD is given (*N* = 3). Asterisks indicate statistical difference between control and KD animals, determined by t-tests with a threshold *P* value of 0.05

The involvement of prostaglandins and the *LsMGST1L*, *LsPGES2* and *LsPGES3L* genes during oxidative stress in salmon louse was further analyzed. The transcript levels of the three putative *PGES* genes in addition to the level of PGE_2_ and PGF_2α_ metabolites were measured in *LsHPX1* KD copepodids previously shown to have increased levels of several oxidative stress-related genes, such as catalase, glutathione reductase, glutathione* S*-transferase (GST) mu and zeta and glutathione peroxidase 1 and 4 [57]. Transcription of *LsMGST1L*, *LsPGES2* and *LsPGES3L* was measured daily in KD animals as the larvae developed from nauplius 2 (3–4 days post hatching [dph]) to copepodids (5–8 dph). The expression of all three transcripts exhibited relatively stable expression in the nauplius stage followed by a peak just after molt to the copepodid stage and a subsequent sharp drop in expression levels at 8 dph (Fig. 7a–c). This drop was not evident in KD animals, and transcription of *LsMGST1L*, *LsPGES2* and *LsPGES3L* was significantly higher in KD animals compared to controls in 8-day old copepodids. In these larvae, the amount of PGE_2_ metabolites and PGF_2α_ was also increased significantly at 8 dph (Fig. 7d, e).

## Discussion

Salmon lice infestations of farmed Atlantic salmon are a major challenge to the industry and hence to future sustainable production. Scientific approaches to understand the interaction between the fish and this resilient ectoparasite has included searches for parasite substances that manipulate the host. Following the identification of PGE_2_ in lice secretory/excretory products, a pivotal role of PGE_2_ as a salmon louse immune-dampening substance was suggested [36] based on its role in immunomodulation by other endo- and ectoparasites [24, 26, 28–30, 67]. However, the exact role of PGE_2_ in the salmon louse host–parasite interaction remains elusive due to technical difficulties obtaining pure secretions from salmon lice glands. Moreover, the presence of additional salmon louse *PGES* genes might have further complicated functional studies. Hence, the aim of the present study was to identify additional salmon louse *PGES* genes, and the bioinformatic analysis of the complete salmon louse genome revealed two additional *PGES* candidates. The results from sequence and phylogenetic analyses support the annotation of LsMGST1L and LsPGES3L, with both sequences well embedded in their respective arthropod clades. However, the enzymatic activity of arthropod PGES has been poorly evaluated; for example, *D. melanogaster* mGST has been found to have GST but not PGES activity [68]. The present study has also not confirmed the enzymatic activity of the putative LsPGESs. Nevertheless, it has, based on present knowledge, identified those genes within the salmon louse genome that have the highest probability of such activity as they are the genes within the genome that show the highest resemblance to characterized *PGES1* and *PGES3* genes of both invertebrates and vertebrates.

Recently, functional studies of the salmon louse *LsPGES2* gene suggested an endogenous role of PGE_2_ because an involvement in the host–parasite interaction could not be demonstrated [40]. The current work was initiated to challenge this role, but instead the results presented here further support these findings. If salmon louse-derived PGE_2_ were to play a major role in host immunomodulation, an upregulation of *LsPGES* transcripts would be expected at the transition from free-living to parasitic stages or when the louse shifts from the attached chalimus 2 stage to the mobile preadult stage. This latter shift allows the lice to move freely over the fish skin, a phase that can be associated with host mortality [6]. However, our expression analysis of *LsMGST1L* and *LsPGES3* during all developmental stages revealed that both transcripts have a stable expression in all developmental stages, with little difference between free-living and parasitic stages, as previously shown for *LsPGES2* [40]. Tissue expression also did not point in the direction of an immune modulative role of salmon louse-derived PGE_2_, as lower expression was detected in samples enriched with exocrine glands and* in situ* hybridization localized *PGES3L* to reproductive organs only. Moreover, KD studies in both copepodids and preadults further supported this conclusion. In mammals, four known PGE_2_ receptors are important for the downstream effects of PGE_2_, eliciting both pro-inflammatory and immunosuppressive responses, particularly in the innate immune cells (reviewed in [69]). This complexity of PGE_2_ signaling is possible not only due to the diversity of receptors that activate different signaling pathways, but also due to different affinities for PGE_2_ and that they are expressed on a variety of cell types in different combinations. This enables PGE_2_ to act as an adaptable signaling molecule in a wide range of cell types. While tick-derived PGE_2_ is believed to have immunosuppressive activity in the host skin [70, 71], PGE_2_ secreted by the enteric amoeba *Entamoeba histolytica* is believed to have a pro-inflammatory effect on colonic epithelial cells [72]. In the present study, a clear but dampened immune response was seen at the site of infestation, especially at the later time point when lice had developed to the chalimus 2 stage. This confirms earlier reports that the immune response to lice in fish skin is mainly limited to a small area surrounding the site of infection [35, 73–75]. Nevertheless, the local level of key pro-inflammatory transcripts expected to be regulated by salmon louse-derived PGE_2_ was induced at the same level in control and dsRNA-treated lice, even though a decrease in *LsMGST1L, LsPGES2* and *LsPGES3L* transcripts of between 75–95% was detected. KD copepodids also successfully infested and developed on Atlantic salmon for 14 days at the same rate as the control lice. Likewise, KD in mobile lice stages targeting all three transcripts did not compromise the ability of the louse to parasitize or reproduce on the fish. Taken together, the presented results indicate that LsMGST1L, LsPGES2 and LsPGES3L play insignificant roles in immunomodulation; alternatively, the concentration of residual proteins was sufficient to maintain normal function through the experimental period.

Earlier studies have demonstrated that the amount of the dopamine-induced release of PGE_2_ in salmon lice secretory/excretory products decreased in the hours after removal from the fish [36], and this has been one of the arguments for an exogenous immunomodulatory role of PGE_2_. The same decrease of PGE_2_ was observed in the present study in whole lice homogenates, especially in lice with a transparent empty-looking gut. Interestingly, lice with a blood-filled intestine at the time of removal did not show the same level of PGE_2_ decrease after 3 h off the host, suggesting that PGE_2_ may be produced during blood digestion. Both salmon skin and blood cells consumed by the louse can represent a source of PGE_2_ as these cells are likely to express all enzymes necessary for PGE_2_ synthesis [11]. Moreover, lysis of blood cells will release toxic components, such as heme and iron, and allow for lipid peroxidation of blood-cell membranes. Thus, digestion of blood leads to an increase in reactive oxidative species, and blood-feeding parasites, including the salmon louse, have evolved mechanisms to handle this stress [76, 77]. Several salmon louse antioxidant genes, such as catalase (EMLSAT00000007315), glutathione reductase (EMLSAT00000000851), GST mu (EMLSAT00000005474) and glutathione peroxidase 1 (EMLSAT00000003186) display relatively high transcript levels within the lice gut (www.licebase.org). *LsMGST1l* also has the highest expression in the lice gut and may act as an antioxidant here.

While non-enzymatic lipid peroxidation typically produces isoprostanes like 8-iso-PGF_2α_, enzymatic lipid peroxidation produces PGE_2_ and especially PGF_2α_ [78, 79]. We therefore wanted to further explore the involvement of the three putative LsPGES in the production of both PGE_2_ and PGF_2α_ metabolites during oxidative stress in the salmon louse. The transcription levels of *LsMGST1L*, *LsPGES2* and *LsPGES3L* increased in copepodids with increased oxidative stress. Accordingly, the amounts of PGE_2_ and PGF_2α_ metabolites were also elevated, indicating a role of prostaglandins in the salmon louse oxidative stress response. This observation is supported by studies of mud crab (*Scylla serrata*) where both lipid peroxidation and antioxidants werre found to vary during the molt cycle [80, 81]. In salmon louse, transcript levels of *PGES* genes, particularly *LsMGSTL*, also varied in different instar ages, as seen for some antioxidant genes, including the above-mentioned catalase, glutathione reductase and glutathione peroxidase 1 [46]. Further studies linking prostaglandins, molt and oxidative stress in salmon louse are thus warranted.

The results of the present study combined with previous findings by [40] also indicate a role of PGE_2_ in salmon louse reproduction. Tissue-specific analysis revealed the highest level of both *LsPGES2* and *LsPGES3L* transcripts in the ovaries and both transcripts are localized to the ovaries and maturing oocytes within the salmon louse genital segment (Fig. 3 and [40]). PGs are well-known to be involved in ovarian development and egg laying behavior in invertebrates (reviewed by [22, 82, 83]), including crustaceans [18, 20, 21]. PGE_2_ is also found to be involved in vitellogenesis in the giant freshwater prawn (*Macrobrachium rosenbergii*), with increased vitellogenin levels in the hemolymph after PGE_2_ administration [18]. Interestingly, *LsPGES3L* transcripts were localized to selected cells of the subepidermal tissue, the site of vitellogenin production in salmon louse [43]. However, while KD of a PGES2 ortholog in the lepidopteran insect *Spodoptera exigua* reduced the egg laying behavior [84], simultaneous KD of *LsPGES2* and *LsPGES3L* did not lead to any measurable decrease in salmon louse reproduction. Furthermore, in animals in which all three putative *LsPGES* genes were targeted, phenotypic alterations were not detected in either reproduction or development, and none of the transcripts were shown to be regulated during the salmon louse reproductive cycle. In the silkworm *Bombyx mori*, egg-laying behavior was not affected when virgin individuals were injected with PGE_2_, showing that PGE_2_ is not an oviposition-stimulating substance of silkworm [85]. The present study suggests a similar situation in salmon louse, with *LsPGES2* and *LsPGES3L* mRNAs possibly only deposited in the salmon louse oocytes for further downstream application after hatching, although PGE_2_ biosynthesis has been confirmed in the ovaries of both the black tiger shrimp (*Penaeus monodon*) and the giant freshwater prawn [18, 21]. It should, however, be mentioned that in *B. mori* and the parasitic nematode *Ascaridia gallia*, a sigma-class GST has been shown to have PGES activity [86, 87], and the presence of additional salmon louse PGES genes could also secure reproduction in KD animals if PGE_2_ has a vital function in salmon louse reproduction. Searches within the salmon louse genome reveal at least 14 genes encoding GSTs (www.licebase.org), although none of them annotates as a sigma-class GST. Nevertheless, the search for additional *PGES* genes in salmon louse should continue to resolve the involvement of PGE_2_ in both endogenous and exogenous processes.

## Conclusion

Putative candidates for immunomodulation of the fish host by salmon louse have attracted interest and speculations, both to understand the molecular interaction but also in the hope of finding candidates for preventive measures. This ectoparasite remains on the fish host for prolonged periods of time with little resulting immunological response. Consequently, it appears prudent to argue that the louse somehow needs to manipulate the host immune response. To date, PGE_2_ has been the main candidate suggested for this role. However, if the present study has in fact succeeded to address all salmon louse PGESs, a role for PGE_2_ as immune modulator seems unlikely. This conclusion is based on three major points: (i) expressional analysis did not indicate a role in immunomodulation; for example, upregulation of transcripts in response to the shift to parasitic stages or high expression in samples with an increased content of exocrine glands were not detected; (ii) functional downregulation of all three transcripts by RNAi did not affect the fish immune response nor the ability of the lice to successfully parasitize the fish; (iii) instead, further experiments supported multiple endogenous roles of PGES in reproduction and in handling of oxidative stress associated with molting and hematophagy. However, the multiple endogenous roles of PGE_2_ together with the presence of multiple PGESs complicates functional studies, and this work could therefore not be regarded as conclusive. Nevertheless, future studies addressing compounds produced by salmon louse exocrine glands are warranted as they are more likely to provide new candidates for immunomodulation of the host.

## Supplementary Information

**Additional file 1.** Sequence alignments and accession numbers used in the phylogenetic analysis of EMLSAT00000006733 in FASTA format, gzipped.

**Additional file 2.** Sequence alignments and accession numbers used in the phylogenetic analysis of EMLSAT00000012943 in FASTA format, gzipped.

**Additional file 3: Figure S1.** Maximum Likelihood phylogenies of orthologues to EMLSAT00000006733 (LsMGST1L) (**a**) and EMLSAT00000012943 (LsPGES3L) (**b**). Branch labels indicate branch support in percentage, scale bars correspond to 0.3 substitutions per site. Sequence annotations are given as found, LsMGST1L = L_salmonis_hPGES in **a**. Both phylograms were rooted using mammalian (**a**) and vertebrate (**b**) sequences as outgroups.

## Data Availability

Data supporting the conclusions of this article are fully available without restriction upon reasonable request.
