# Supplementary figures and images for: Roles of three putative salmon louse (Lepeophtheirus salmonis) prostaglandin E2 synthases in physiology and host–parasite interactions
Source: Parasit Vectors. 2021 Apr 19;14:206. doi: 10.1186/s13071-021-04690-w (PMC8056522; doi:10.1186/s13071-021-04690-w)

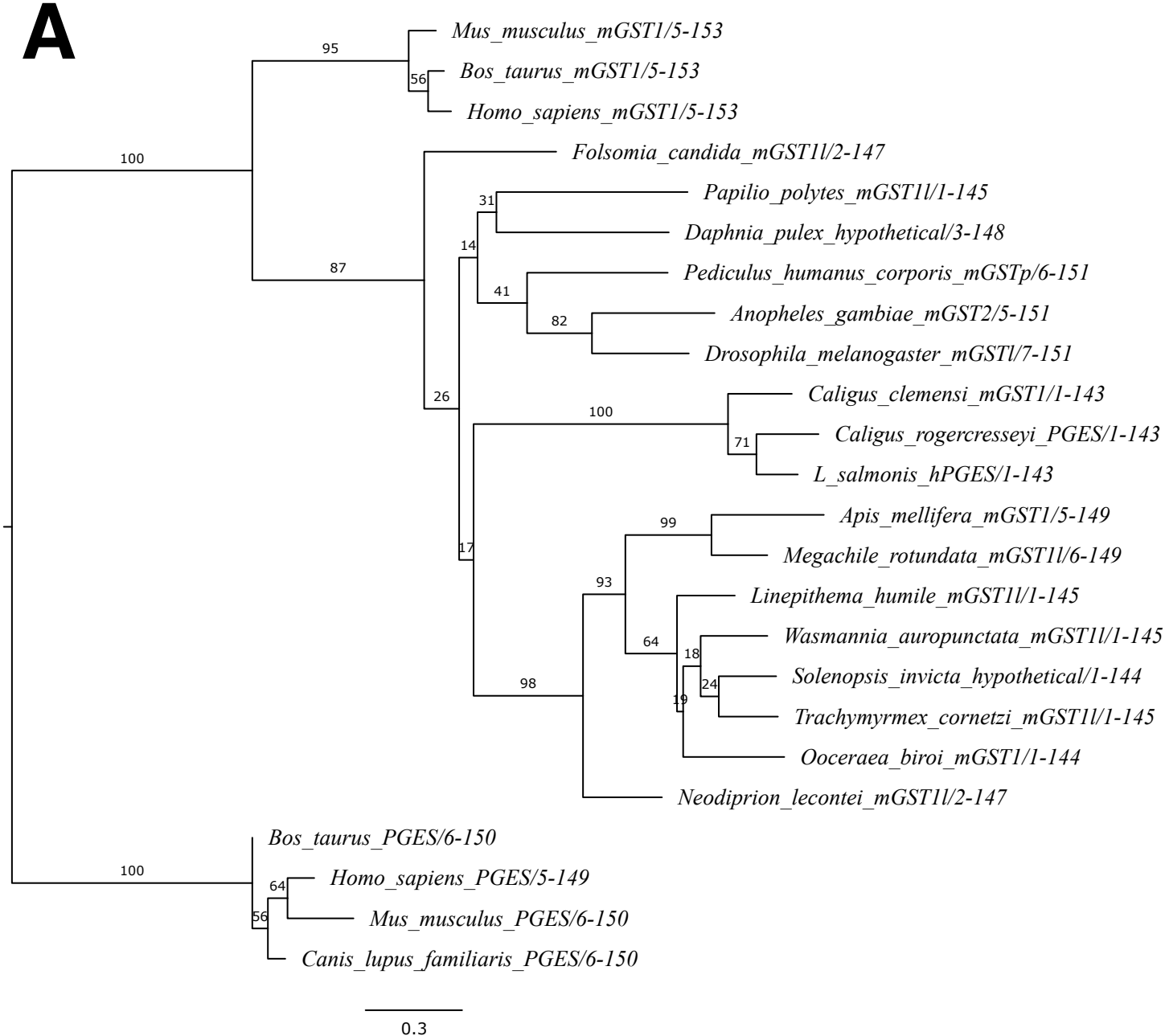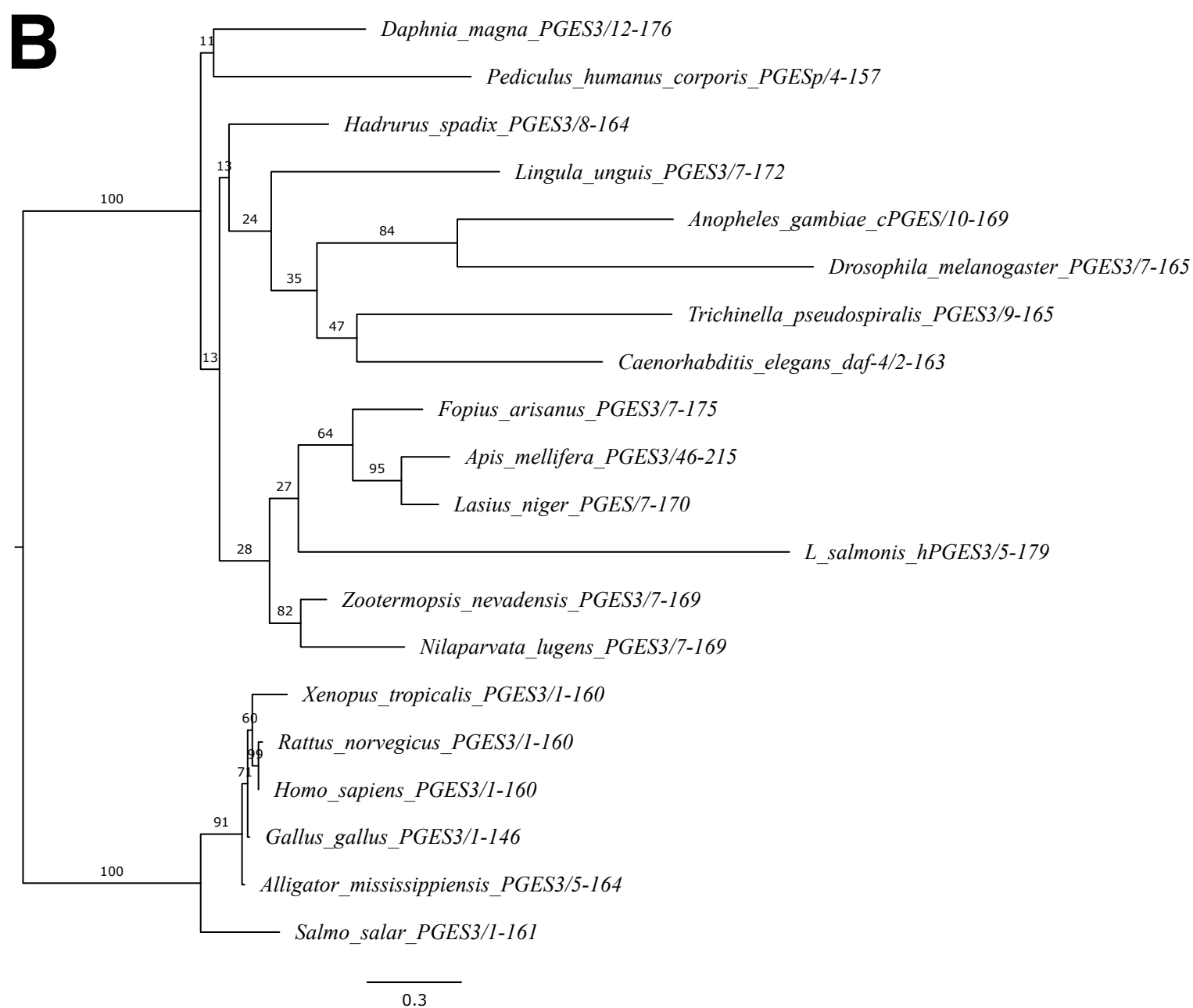

Supplement: Supplementary file 3 — Additional file 3: Figure S1. Maximum Likelihood phylogenies of orthologues to EMLSAT00000006733 (LsMGST1L) (a) and EMLSAT00000012943 (LsPGES3L) (b). Branch labels indicate branch support in percentage, scale bars correspond to 0.3 substitutions per site. Sequence annotations are given as found, LsMGST1L = L_salmonis_hPGES in a. Both phylograms were rooted using mammalian (a) and vertebrate (b) sequences as outgroups. [file 13071_2021_4690_MOESM3_ESM.pdf]
